# Development of a Physiological Frailty Index for the World Trade Center General Responder Cohort

**DOI:** 10.1155/2018/3725926

**Published:** 2018-02-26

**Authors:** Ghalib A. Bello, Roberto G. Lucchini, Susan L. Teitelbaum, Moshe Shapiro, Michael A. Crane, Andrew C. Todd

**Affiliations:** Department of Environmental Medicine and Public Health, Icahn School of Medicine at Mount Sinai, New York, NY, USA

## Abstract

Responders to the 9/11/2001 WTC attacks were exposed to multiple toxic pollutants. Since 2002, the health of the responder cohort has been continuously tracked by the WTC Health Monitoring Program. However, no assessments have been made of frailty, an important health metric given the current average age of the WTC responder cohort (55 years). In this study, we use laboratory test results and other physiological parameters to construct a physiological frailty index (FI-Lab) for this cohort. The study sample comprised responders aged 40 years or older who completed a health monitoring visit at Mount Sinai Center within the past 5 years. For each subject, FI-Lab was computed as the proportion of 20 physiological parameters (lab tests, pulmonary function, and blood pressure) on which the subject had abnormal values. Using negative binomial regression models, we tested FI-Lab's association with the SF-12 wellbeing score and various demographic characteristics. FI-Lab showed strong associations with the physical and mental components of the SF-12 as well as age, race, and smoking status. Using a cutoff of 0.25 to define presence of physiological/preclinical frailty, we found frailty prevalence in the study sample to be approximately 12%. This study demonstrates the feasibility of assessing preclinical frailty in the WTC responder cohort.

## 1. Introduction/Background

In the years following the terrorist attacks of 9/11/2001, efforts have been made to monitor the health of rescue and recovery workers involved in the emergency response and subsequent cleanup efforts. A cohort of WTC 9/11 general (nonfirefighter) responders (the General Responder Cohort (GRC)) has been established. As this cohort ages, characterization of the changes in health patterns due to aging is becoming increasingly important. The dynamics of the aging process vary considerably across individuals in any population, which is relevant to understanding anticipated changes in physical health and cognitive functioning [1–3]. Evidence of this heterogeneity can be observed from the molecular/cellular level (via telomere dynamics, DNA methylation patterns, etc.) up to the macroscopic level (frailty, mortality, etc.) [4]. The aim of this study was to evaluate age-related deterioration in physiological functions using the clinical construct of* frailty* [5]. Frailty is a physical state characterized by increased vulnerability to adverse health outcomes and is believed to arise from diminishing physiological reserve and gradual loss of the body's ability to maintain homeostatic equilibrium [6]. It has become recognized as a standard geriatric syndrome [7] and there have been increased calls for including frailty assessment as a part of routine clinical encounters [8].

Since 2002, the World Trade Center Health program (WTCHP) has enrolled responders and conducted clinical and health monitoring on this cohort (which continues to increase) [9]. Frailty screening/assessment has not been part of these health evaluations, but the clinical data collected on this cohort contains elements that can be used to measure frailty. Due to its clinically complex profile, no consensus definition of frailty currently exists. In this study, we adopted one popular approach, introduced by Mitnitski et al. (2001) [10], which conceptualizes frailty as the accumulation of functional and health deficits resulting from (and indicative of) a diminishing ability to maintain normal function/homeostasis [11]. With this “deficit accumulation” model, frailty is measured by computing the proportion of considered deficits present in an individual [12]. The considered deficits typically span multiple domains of health and wellbeing: disability, functional impairment, health conditions, laboratory test abnormalities, diseases, and so forth. The proportion present in an individual is referred to as a frailty index (FI), for which higher scores indicate greater proportion of age-related health problems. The FI approach is particularly useful for the 9/11 responder cohort as it offers a considerable degree of latitude in the choice of variables considered for FI [13]; the variables can be selected from any available pool of health-related measures (clinical databases, electronic medical records, etc.) and typically integrate multiple domains of aging-related health issues, for example, reduced mobility and strength, presence of comorbidities, polypharmacy, reduced physical activity, disabilities, poor self-rated health, problems with activities of daily living, and physical or neurological/cognitive symptoms. Since the introduction of the cumulative deficit model of frailty, various FIs have been developed, but consistent patterns have emerged, with several studies reporting that FIs are better predictors of adverse health outcomes than chronological age [14]. While most FIs typically use observable clinical deficits in health, physical functioning, and so forth, the recent years have seen the emergence of a new class of FIs that are based solely on standard laboratory test biomarkers. These biomarkers are often clinical chemistry lab test results measured in routine diagnostic panels: cholesterol, creatinine, blood glucose, serum potassium, and so forth. [15]. Abnormalities in the levels of these physiological parameters are linked to aging-related dysregulation in multiple organ systems [16]. This type of frailty index (generally referred to as FI-Lab [15]) is computed by determining the number of biomarkers/physiological parameters on which an individual falls outside of the normal/reference range. Since its introduction, multiple studies have shown that FI-Lab exhibits good agreement with the “clinical FIs” that are based largely on clinical deficits [17]. FI-Lab demonstrates strong predictive power for mortality, frequency of hospital utilization, polypharmacy, and self-assessed health status [15, 17–21]. A recent study showed that FI-Lab is also associated with telomere length [22]. The use of laboratory parameters and examination data potentially makes FI-Lab a more objective measure than the commonly used clinical FIs, which often rely heavily on subjective self-report of functional impairments [17].

Using year 2012 as a starting point (approximately a decade after 9/11), this study evaluated and characterized preclinical frailty among WTC cohort members aged 40 years and older. The specific aims of the study were to (1) develop a* WTC FI-Lab* from physiological parameters obtained from laboratory tests and anthropometric and spirometric measures collected during WTCHP clinical monitoring visits, (2) examine the validity of WTC FI-Lab by assessing its relationship to an established instrument for self-assessed health, and (3) test the association of WTC FI-Lab with demographic and functional characteristics of the GRC.

## 2. Methods

### 2.1. World Trade Center General Responder Cohort

Enrollment in the WTC Health Program (WTCHP) requires meeting the following eligibility criteria: (1) handled and/or processed human remains as a member of the New York City Office of Chief Medical Examiner, (2) worked for the Port Authority Trans Hudson (PATH) Corporation and during the period between February and July 2002 spent more than 24 hours cleaning PATH tunnels, and (3) participated in the 9/11 cleanup effort for at least 4 hours anytime during the period of 11–14 September 2001 or for at least 24 hours in the month of September 2001 or for a total of at least 80 hours in the 10 months following the attacks [9].

As part of the WTCHP, enrollees have periodic (often annual) clinical monitoring visits at which they receive a battery of health assessments. Our study utilized clinical data collected during these monitoring visits.

### 2.2. Sample Selection

The goal of this study was to assess preclinical frailty among the WTC GRC, starting from approximately 10 years after 9/11 attacks in 2001. We selected GRC members who had at least one follow-up clinical monitoring visit in 2012 or later and who were 40 years old or older at the time of the visit. Our study was restricted to members whose monitoring visits were conducted at the largest WTCHP clinic, Mount Sinai Selikoff Centers for Occupational Health in New York City, because few clinical chemistry lab results were available for responders attending any other clinic. Any visits with missing clinical diagnostic and health outcome variables were excluded from consideration. For the purposes of these analyses, only the most recent visit (hereafter “index visit”) of each subject was considered.

### 2.3. Derivation of WTC FI-Lab

WTC FI-Lab was constructed from diagnostic data collected on GRC members at clinical monitoring visits. The data were obtained from standard laboratory blood tests, physical examinations, and pulmonary function tests. Physical examinations involved measurement of anthropometric parameters such as weight, height, body mass index (BMI), blood pressure, and heart rate. Pulmonary function tests assessed lung function using spirometric measures, Forced Expiratory Volume in 1 second (FEV_1_) and Forced Vital Capacity (FVC).

All physiological parameters were measured on continuous scales (e.g., blood pressure in units of mmHg) but for the purposes of computing FI-Lab, they were dichotomized based on standard clinical reference ranges. This involved recoding each physiological parameter into a binary variable representing a normal or abnormal result. Normal (within reference range) results were recoded as “0” and abnormal (outside reference range) results were recoded as “1.” For example, a commonly used clinical reference range for systolic blood pressure (SBP) is 90–140 mmHg. Values of SBP within this range are considered “normal,” and values outside this range are considered “abnormal.” If a subject had a systolic blood pressure measure of 160 mmHg, this was recoded as “1,” because it falls outside of the normal range of SBP and was considered a “deficit,” indicative of the presence of increased risk due to abnormal SBP. An individual's FI-Lab score was then computed by summing the dichotomized variables across the physiological parameters and expressing this score as the proportion of deficits across all physiological parameters considered, yielding a value between 0 and 1 [15].

Physiological parameters used to construct FI-Lab need to be good proxy measures for the dysregulation that occurs with aging. In this study, selection of physiological parameters for inclusion in the WTC FI-Lab was carried out in a systematic fashion, following a widely used protocol outlined by Searle et al. [13] for constructing frailty indices. According to the Searle protocol, a variable/item is considered appropriate for inclusion in a frailty index if it is associated with health status and if the prevalence of deficits (abnormal values) on this variable increases with age. Another recommendation is that the set of variables/items used to construct a frailty index should span multiple dimensions/domains of health [13, 23]. In the context of FI-Lab construction, we interpreted this to mean that the set of physiological parameters chosen for inclusion in the index should span multiple organ systems, thereby making the index a global/holistic measure of physiological function. Candidate variables for inclusion in the FI-Lab were diagnostic measures from routine laboratory blood tests, physical exam, and the pulmonary function tests (the full list is provided in Table 1). The proportion of individuals with abnormal values was calculated for ages of 40 to the maximum age in the study sample in 1-year increments. These proportions were plotted against age to examine how the prevalence of abnormal test results varied with age. Spearman coefficient of the correlation between age and the prevalence of abnormal values was computed for each candidate physiological parameter. Parameters with correlation ≥ 0.4 (i.e., at least moderate positive correlation with age) were selected for inclusion in the WTC FI-Lab. The final set of parameters was reviewed to make sure there was sufficient representation of various organ systems.

### 2.4. Validation of WTC FI-Lab and Assessment of Association with Cognitive Impairment

We examined the association of FI-Lab with concurrent self-reported health/wellbeing measures from a questionnaire administered during clinical monitoring visits. This questionnaire (the Self-Administered Mental Health Questionnaire [SAMHQ]) solicits, among other things, information about general wellbeing, which is assessed using items from the SF-12©, a validated survey designed for self-assessment of physical and mental/emotional health [24]. This 12-item questionnaire assesses overall quality of life via questions about physical impairments, limitations in ability to perform activities of daily living due to physical pain, emotional problems, and general level of energy and vitality. Responses to these items are used to compute two summary scores: the Physical Component Score (PCS) and the Mental Component Score (MCS). The PCS and MCS range from 0 to 100, with higher values indicating better physical/mental wellbeing. We used multivariable regression to determine the association of WTC FI-Lab with SF-12 PCS at the index visit, adjusting for primary characteristics of the cohort: age at index visit, sex, race/ethnicity, pre-9/11 occupation, education, year of WTCHP enrollment, WTC exposure severity, year of index visit, and smoking status at index visit (details on these covariates are provided in the next section). A similar regression model was used to assess the covariate-adjusted association of WTC FI-Lab with MCS. In both these models, the outcome/dependent variable was WTC FI-Lab, treated here as a count variable (the raw count of deficits). A Poisson regression approach and a negative binomial regression approach were both considered, as these are the standard methods for modeling count outcomes [25]. We chose negative binomial regression because it is more flexible and naturally handles overdispersion [26]. A significance level of 5% was used.

The SAMHQ also assesses self-reported problems with short-term memory [9]. One item on the SAMHQ asks respondents to report the frequency of problems with short-term memory, for example, forgetting keys or grocery store items. The response is a multiple-choice, five-point Likert scale indicating the degree of memory problems: (1) “not at all,” (2) “a little bit,” (3) “moderately,” (4) “quite a bit,” and (5) “extremely.” For the purposes of this analysis, we collapsed this five-point Likert scale into two aggregate response categories, (1) “not at all/a little bit” and (2) “moderately/quite a bit/extremely,” yielding a binary variable. Logistic regression was used to test the association of WTC FI-Lab with this binary variable (at the index visit), adjusting for age at index visit, sex, race/ethnicity, pre-9/11 occupation, education, year of WTCHP enrollment, WTC exposure severity, year of index visit, and smoking status at index visit. Effect size was quantified using odds ratios with 95% confidence intervals.

### 2.5. Association of WTC FI-Lab with Cohort Characteristics

We tested the association of FI-Lab with the following demographic and exposure characteristics of the general responder cohort: age at index visit, sex, race/ethnicity, pre-9/11 occupation, educational attainment, WTC exposure severity, year of WTCHP enrollment, year of index visit, and smoking status at index visit. Data on date of birth, sex, race, and pre-9/11 occupation were collected at the time of WTCHP enrollment [9].

For each cohort member, WTC exposure severity was assessed via an Exposure Assessment Questionnaire (EAQ), designed to evaluate the extent of exposure to pollutants prior to and while working on the rescue and recovery effort. Responses to the EAQ were used to construct an exposure severity variable with four severity levels: low, intermediate, high, and very high exposure. These categories were defined based on a cohort member's duration of work on the WTC cleanup effort, exposure to the dust cloud on 9/11, and whether or not they worked on the debris pile (complete details are available in [27]). In this study, we merged the “high” and “very high” exposure categories into one category. Smoking status at index visit was determined from responses to the Interviewer-Administered Medical Questionnaire (IAMQ). Administered to GRC members at every monitoring visit, the IAMQ gathers information such as demographics, history of tobacco and alcohol use, employment status, and current/prior medical conditions (self-reported) [9].

The association of WTC FI-Lab with each of the aforementioned cohort characteristics was assessed via a negative binomial regression model treating FI-Lab (as deficit count rather than proportion) as the outcome/dependent variable and all cohort characteristics as independent variables. This multivariable regression approach allowed us to assess the relationship of each cohort characteristic with FI-Lab while adjusting for the effects of other characteristics.

### 2.6. Estimating Prevalence of Frailty in the General Responder Cohort

WTC FI-Lab was used to estimate the prevalence of frailty in our study sample. As mentioned earlier, the raw score on this index (count of deficits) may be represented as the proportion of physiological parameters for which a subject is in deficit. We dichotomized the FI (as a proportion) using a cutoff of 0.25, a commonly used threshold for classifying patients as frail versus nonfrail [28, 29], and estimated the percentage of our study sample classified as frail. We also estimated the prevalence of frailty among strata of various cohort characteristics (age group, sex, race, etc.). For each cohort characteristic, a chi-square test was used to assess the statistical significance of observed differences in frailty prevalence across strata of the characteristic. Continuous characteristics like age and year of WTCHP enrollment were appropriately discretized. Age was divided into the following groups: 40–45, 46–50, 51–55, 56–60, 61–65, and >65 years. WTCHP enrollment year was discretized into the following categories: 2002–2005, 2006–2008, and 2009–present.

Negative binomial regression models were run using the GENMOD procedure in SAS® 9.4 (Cary, NC). Logistic regression models were run in SAS procedure LOGISTIC. Plots were generated with SAS and R [30]. All analysis was carried out at the 5% significance level.

## 3. Results

### 3.1. Summary Statistics on Study Sample

A total of 9,329 subjects met the sample selection criteria. Of these, 524 were excluded due to missing values on the variables used to construct the index. Further, 927 subjects were excluded due to missing values on outcome variables (SF-12 PCS and MCS), and an additional 532 were excluded due to missing values on demographic characteristics. This yielded a final sample of 7,346. No significant differences in demographic characteristics and outcome distributions were observed between the excluded subjects and those remaining in the final sample.  Table 2 summarizes demographics of the WTC GRC members included in the final study sample (*n* = 7,346). The median age on 9/11/2001 was 39 years. At the index visit, ages ranged from 40 to 85 (with a median age of 51 years). A majority of the study sample (97.6%) were aged 70 and younger. The majority of these individuals were male (~83%), non-Hispanic Caucasian (54%) and in protective services (e.g., law enforcement) or construction-related occupations prior to 9/11 (~71%). Nearly half of these individuals enrolled in the WTCHP (and completed their first monitoring visit) between 2002 and 2005. And based on responses to the Exposure Assessment Questionnaire, WTC exposure severity in most of these individuals was ranked as either intermediate (65%) or high/very high (21%). In our study sample, the majority of individuals (~91%) had their index visit between 2012 and 2014. At the index visit, 61% of study participants reported never having smoked.

### 3.2. Selection of Parameters for Inclusion in WTC FI-Lab

Of 33 candidate physiological parameters (see Table 1), 20 met the FI-Lab selection criteria outlined in Methods. These parameters are listed in Table 3, along with corresponding reference ranges and summary statistics on measured values within our study sample. Collectively, these 20 parameters gauge the functional status of various physiological systems, for example, hematological function (white blood cell count, platelet count, lymphocyte count, etc.), lung function (FEV_1_ and FVC), cardiovascular function (blood pressure and heart rate), kidney function (creatinine and blood urea nitrogen), liver function (albumin), and metabolic function (glucose and electrolytes).

**Figure 1:**
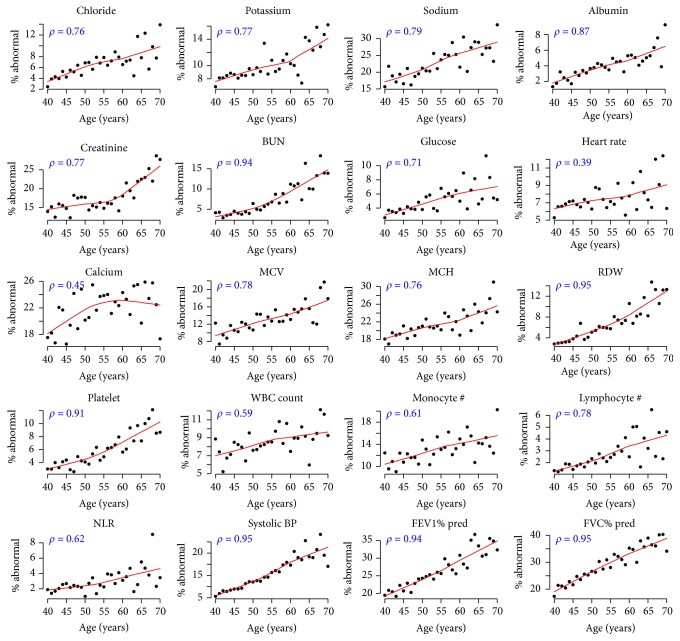
Plots depicting the rate of abnormal values of physiological parameters over ages 40–70. Each plot corresponds to one physiological parameter, with black dots representing raw data (% of individuals of a certain age with abnormal values on a physiological parameter). Smooth fits (solid red curve) were computed using locally weighted scatterplot smoothing (LOESS). Spearman correlation is provided on the top left corner of each plot. BUN: blood urea nitrogen; MCV: mean corpuscular volume; MCH: mean corpuscular hemoglobin; RDW: red cell distribution width; WBC: white blood cell; NLR: neutrophil-to-lymphocyte ratio.

Figure 1 shows plots depicting the age-dependence of the rate of deficits (abnormal values) on each of the 20 FI-Lab physiological parameters. The plots show that, for all 20 of these parameters, the probability of falling outside of the “normal” (reference) range generally increases with age, reflecting the progressive loss of the ability to regulate the levels of these parameters. Each plot also shows the Spearman coefficient of the correlation between age and the prevalence of abnormal values. While the full age range (at index visit) within our study sample was 40–85, these plots used a range of 40–70 because of sparse data beyond age of 70 (only 2.4% of subjects in our study sample were over the age of 70 at the index visit).

**Figure 2:**
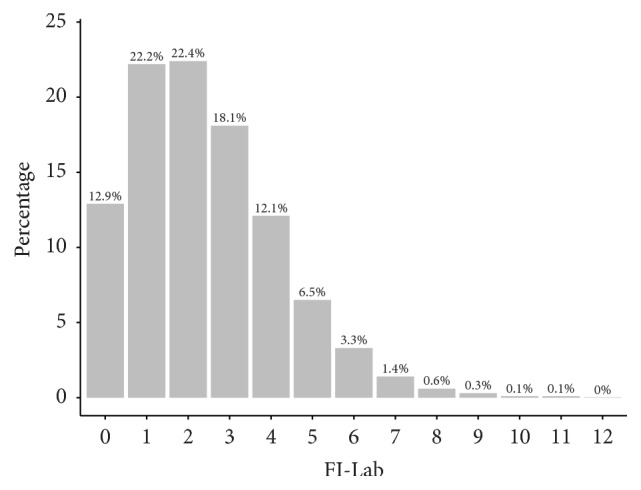
Distribution of FI-Lab across study sample.

Figure 2 is a bar-plot depicting the distribution of the computed FI-Lab values (represented here as deficit counts) across the study sample. The distribution shows the characteristic, right-skewed pattern of frailty indices, with many individuals having few or no deficits [31]. In our study sample, ~13% of subjects had zero deficits; that is, they had normal values (within reference range) on all 20 physiological parameters comprising the FI-Lab. An additional ~44% had 1-2 deficits, and ~30% had 3-4 deficits. The maximum observed number of deficits was 12 out of 20 (60% deficit rate). It is noteworthy that this observed maximum is close to the “universal limit” of deficits (70%) [14]. It has been demonstrated that, for frailty indices, the probability of having deficits on more than 70% of index components is nearly zero, and this 70% limit has been proposed as a biologically relevant transition point beyond which survival (and further deficit accumulation) is not sustainable [32]. This limit has been consistently observed across several studies on frailty [33–37].

### 3.3. Association of FI-Lab with SF-12 and Self-Reported Short-Term Memory Problems

**Figure 3:**
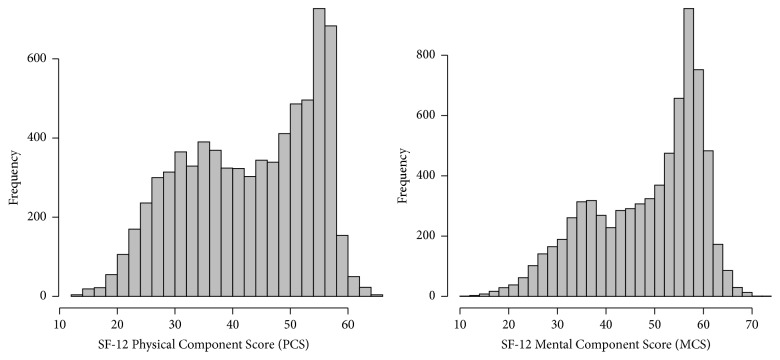
Distribution of SF-12 PCS and MCS in study sample.

Figure 3 depicts histograms of the physical (PCS) and mental (MCS) components of the SF-12 questionnaire scores within our study sample. The median PCS was 44.3 (IQR: 33.5–53.1), and the median MCS was 51.7 (IQR: 39.5–57.7). While the SF-12 PCS and MCS are designed to have a possible range of 0–100, in our study sample, the observed ranges were 12.2–65.6 and 10.4–72, respectively.

In negative binomial regression models, PCS showed a significant negative association with FI-Lab (beta coefficient = −0.0092, *p* < .0001), as did MCS (beta coefficient = −0.0044, *p* < .0001), after adjusting for cohort characteristics. These results imply that increasing PCS/MCS is associated with lower FI-Lab. Since the PCS and MCS are scored in such a way that higher values correspond to greater physical/mental wellbeing [24], our results indicate that FI-Lab score is inversely correlated with degree of physical/mental wellbeing; that is, those with more deficits report lower physical and mental wellbeing, and vice versa.

Table 4 summarizes responses to the SAMHQ item on self-reported degree of short-term memory problems. Within our study sample, ~55% of subjects reported experiencing memory problems rarely or never experiencing memory problems, while 45% reported experiencing these problems moderately or worse. Our analysis revealed that these two groups differed significantly on FI-Lab. The logistic regression model showed that, after adjusting for cohort characteristics, the effect of FI-Lab was significant (*p* < 0.0001), with odds ratio of 1.06 (95% CI: 1.027–1.086), indicating that, for every additional deficit, the odds of reporting short-term memory problems increase by 6%.

### 3.4. Association of FI-Lab with Cohort Characteristics

Negative binomial regression models were used to examine the association of FI-Lab deficit count with age at index visit, sex, race/ethnicity, pre-9/11 occupation, education, year of WTCHP enrollment, WTC exposure severity, year of index visit, and smoking status at index visit. The results are summarized in Table 5. Age at index visit showed a significant positive association with the FI-Lab deficit count (*p* < 0.0001), indicating that older individuals had higher FI-Lab score. Sex showed a significant association with FI-Lab (*p* = 0.0157), with females exhibiting fewer deficits. Relative to Caucasian subjects, black subjects had significantly higher FI-Lab (*p* < 0.0001), as did Hispanics (*p* = 0.0095). Current smokers had significantly higher FI-Lab than those who had never smoked (*p* < 0.0001). No significant associations were seen for pre-9/11 occupation, education, WTCHP enrollment year, WTC exposure severity, or year of index visit.

### 3.5. Assessment of Frailty Prevalence

Computed values of WTC FI-Lab (expressed as deficit proportions) were recoded into binary values using a cutoff of 0.25 (deficits on five of the 20 physiological parameters comprising the WTC FI-Lab). Subjects with FI-Lab equal to or exceeding this threshold were classified as frail. In our study sample, 12.3% of the 7,346 subjects fell into the frail category. Estimated frailty prevalence (% with FI-Lab ≥ 0.25) for categories of various cohort characteristics are summarized in Table 6 (with corresponding *p*-values). Frailty prevalence increased with successively older age groups (*p* < 0.0001), showing a nearly fourfold increase from the lowest age group (40–45) to the highest (>65). Frailty was marginally less prevalent in females (*p* = 0.052). Frailty rate was highest in blacks (*p* < 0.0001) and roughly equal among other racial groups. GRC members in protective occupations (pre-9/11) had noticeably lower frailty rates compared to those in other occupation categories (*p* < 0.0001). Those with high school education or lower had higher frailty rates compared with those with greater than high school education (*p* = 0.0096). Current smokers had a significantly higher frailty rate than former or never smokers (*p* < 0.0001). No significant variations in frailty prevalence were seen across levels of WTC exposure severity, enrollment year, or year of index visit.

## 4. Discussion

In this study, we developed and tested a frailty index constructed from physiological parameters (routine laboratory tests and anthropometric measures). This class of frailty indices is gaining popularity as an alternative to clinical FIs. The key distinction between clinical FI and FI-Lab is that the latter is based entirely on biochemical markers/physiological parameters; hence it is believed to represent the burden of preclinical or subclinical deficits [18]. Subclinical deficits can be thought of as systemic, organ-level dysregulation that occurs as a direct result of molecular or cellular level damage (e.g., oxidative stress and telomere attrition) and in turn leads to “macroscopic” (clinically evident) functional deficits and impairments such as disease, weakness, limited mobility, cognitive changes, and sensory loss [4]. In some sense, the subclinical dysregulation measured by FI-Lab provides an intermediate link within cellular-level damage that eventually scales up to clinically detectable impairments/deficits [17]. We therefore believe that WTC FI-Lab has the potential to identify frailty in its early stages among our cohort. In accordance with this, we used a relatively low age cutoff in the selection of the sample for this study. As a result, our study cohort is younger (median age of 51) than those in other frailty studies, which often focus on the elderly. The rationale behind this sample selection choice is that the development of WTC FI-Lab and other biomarker-based tools for biological age assessment will allow screening of those at risk of advanced frailty endpoints. There is a growing evidence that frailty may be modifiable/reversible, especially in the early stages [38] preceding the onset of more advanced symptoms. This calls for the development of valid tools for screening at-risk individuals.

The WTC FI-Lab was developed using a standard protocol for the construction of frailty indices [13]. A number of candidate physiological parameters were screened, and 20 were deemed suitable for inclusion in the index. These parameters all showed significant age-sensitivity; that is, the probability of having abnormal values on each parameter increased with age. Collectively, the parameters selected for WTC FI-Lab span multiple domains of physiological/organ function: respiratory, cardiovascular, hematologic, and metabolic function. Our FI-Lab differs in composition from other FI-Lab measures [15, 17–21]. This is normal, as most frailty indices are designed based on whatever data are available to researchers. Empirical investigations have demonstrated that the composition of items included in a frailty index has relatively little effect on its aggregate properties [39]. As long as a sufficiently large set of valid items is used, the general characteristics of FI are fairly robust to the specific composition of items [39, 40].

We attempted to validate WTC FI-Lab by evaluating whether it is associated with an established instrument for assessing physical and mental health: the SF-12 [24]. This survey is one of the most commonly used instruments for assessment of general health [41]. It has shown associations with a range of adverse aging-related health outcomes, including mortality and hospitalization [42, 43]. A recent study found that the SF-12 is also associated with frailty [44] and a number of previous studies have actually used SF-12 survey items to construct frailty indices and frailty phenotype measures [45–47]. It has also exhibited association with mitochondrial DNA copy number, a biomarker of aging [48]. These properties make the SF-12 a useful instrument for assessing construct validity of the WTC FI-Lab. We found that both the physical and mental component subscores of the SF-12 are associated with WTC FI-Lab, after adjusting for several cohort characteristics. These results indicate that the physiological dysregulation measured by WTC FI-Lab shows good agreement with concurrent self-assessments of physical and mental health.

Posttraumatic stress disorder (PTSD) has been observed among a fraction of the WTC responder cohort and has been linked to onset of dementia and cognitive impairment [49]. Since frailty is known to be associated with late-life cognitive decline [50–53], we were interested in evaluating the relationship of WTC FI-Lab with a single-item measure of self-assessed memory problems. Logistic regression analysis showed that higher scores on FI-Lab are associated with higher odds of reporting moderate/severe short-term memory problems and that this effect was independent of age, sex, race/ethnicity, education, and other relevant covariates.

We assessed the relationship of FI-Lab with various cohort characteristics and observed associations with age, sex, race, and smoking status. The association with age is expected and serves as a further confirmation of the construct validity of WTC FI-Lab. The association of FI-Lab with sex was due to lower values observed in female cohort members. This trend has also been observed in a population study on another lab-based frailty index [15]. The strong association observed for race/ethnicity in our cohort was driven largely by higher scores in African Americans. This finding is in line with multiple epidemiological studies that identify African-American background as a significant and independent risk factor for frailty [54–56]. It is worth noting that severity of exposure on 9/11 and during the subsequent cleanup effort (assessed by a three-level severity index) showed no association with FI-Lab. We note, however, that the exposure index we used has limited accuracy in capturing the scope and severity of 9/11 exposure. In particular, it does not explicitly incorporate the effect of psychological trauma, a factor that has been linked with accelerated aging [57, 58].

One limitation worth highlighting is the extent to which the WTC FI-Lab was validated in this study. A key step in the development of a frailty index is assessment of its validity as a measure of the latent construct of frailty [59]. It is commonplace to consider three major facets of validity: content validity, construct validity, and criterion validity [59]. Content validity of WTC FI-Lab is evident from the systematic procedure used in its construction: we selected age-sensitive biomarkers that are indicative of organ and physiological dysfunction, major features of aging. Construct validity of WTC FI-Lab was assessed via convergent construct correlation [60], showing that it is associated with the SF-12, a validated and established instrument for assessing physical and mental wellbeing. Criterion validity for FIs is defined as the ability to predict adverse health outcomes, typically mortality [61]. Currently, mortality data are not available on cohort members beyond December 2011 [62], so we were unable to evaluate the predictive power of WTC FI-Lab for mortality.

We have developed a laboratory test-based frailty index for the WTC general responder cohort. Validation analyses showed that the WTC FI-Lab shows a strong association with the SF-12, an established measure of physical and mental wellbeing (SF-12). We also found a putative association with self-reported short-term memory problems among our cohort. Future work will focus on further validation of the index, particularly via collection of up-to-date mortality data on the cohort.

## Figures and Tables

**Table 1:** Candidate physiological parameters considered for inclusion in WTC FI-Lab.

Systolic blood pressure
Diastolic blood pressure
Heart rate
Glucose
Chloride
Calcium
Sodium
Potassium
Triglycerides
Total cholesterol
Creatinine
Blood urea nitrogen
White blood cell count
Neutrophil count
Eosinophil count
Basophil count
Lymphocyte count
Monocyte count
Albumin
Total protein
Alkaline phosphatase
Aspartate aminotransferase (AST)
Alanine aminotransferase (ALT)
Total bilirubin
Forced expiratory volume in 1 s (FEV_1_) (% predicted)
Forced vital capacity (FVC) (% predicted)
Platelet count
Red cell distribution width
Mean platelet volume
Red blood cell count
Hemoglobin
Mean corpuscular volume
Mean corpuscular hemoglobin

**Table 2:** Summary of demographic and exposure characteristics of the study sample.

Characteristics	Summary statistics
Sample size	7346
Age at 9/11 (median, IQR, range)	39 (34–45, 25–74)
Age at index visit (median, IQR, range)	51 (46–57, 40–85)
Sex	
Female	1229 (16.7%)
Male	6117 (83.3%)
Race/ethnicity	
White	3983 (54.2%)
Black	1084 (14.8%)
Hispanic	2100 (28.6%)
Other	179 (2.4%)
Smoking status at index visit	
Never	4484 (61.0%)
Former	2289 (31.2%)
Current	573 (7.8%)
Pre-9/11 occupation	
Construction	1781 (24.2%)
Protective	3466 (47.2%)
CM and IRG^*∗*^	709 (9.7%)
Other	1390 (18.9%)
WTCHP^†^ enrollment year	
2002–2005	3594 (48.9%)
2006–2008	2296 (31.3%)
>2008	1456 (19.8%)
Index visit year	
2012	1574 (21.4%)
2013	2399 (32.7%)
2014	2693 (36.7%)
2015	468 (6.4%)
2016	212 (2.9%)
Educational attainment	
High school or less	2263 (30.8%)
>High school	5083 (69.2%)
Exposure severity	
Low	990 (13.5%)
Intermediate	4793 (65.2%)
High/very high	1563 (21.3%)

^*∗*^CM: cleaning/maintenance of buildings and grounds; IRG: installation/repair groups (electrical, telecommunications, and others); ^†^WTCHP: WTC Health Program.

**Table 3:** Median (IQR) measured at index visit for study sample (*n* = 7,346).

Physiological parameter	Reference range	Median (IQR)
Chloride (mEq/L)	96–106	103 (101–105)
Potassium (mEq/L)	3.8–5	4.4 (4.1–4.6)
Sodium (mEq/L)	136–142	141 (139–142)
Albumin (g/dl)	>3.8	4.4 (4.2–4.6)
Creatinine (mg/dl)	0.6–1.2	1.04 (0.92–1.16)
Blood urea nitrogen (mg/dl)	8–23	16 (14–19)
Glucose (mg/dl)	70–200	94 (86–105)
Heart rate (beats/minute)	60–100	68 (64–76)
Calcium (mg/dL)	9.2–10.8	9.5 (9.2–9.7)
Mean corpuscular volume (fL)	80–96	90.3 (87.3–93)
Mean corpuscular hemoglobin (pg)	27–32	30.4 (29.2–31.4)
Red cell distribution width, SD (%)	≤13.7	12.2 (11.7–12.7)
Platelet count (×10^3^/*μ*L)	150–450	222 (191–258)
White blood cell count (×10^9^/L)	4.3–10.8	6.6 (5.5–7.8)
Monocyte count (×10^9^/L)	0.3–0.7	0.5 (0.4–0.6)
Lymphocyte count (×10^9^/L)	1–4.5	1.9 (1.5–2.2)
Neutrophil : lymphocyte ratio	<5	2.1 (1.6–2.75)
Systolic blood pressure (mmHg)	90–140	124 (116–134)
FEV_1_ (% predicted)	>80	89.6 (79.9–98.9)
FVC (% predicted)	>80	87 (78.4–95.5)

**Table 4:** Frequency table of responses to SAMHQ item on short-term memory problems.

How much trouble have you had with your short-term memory (e.g., forgetting where you left keys, grocery store items, etc.)?		
Response	Number	Percentage
Not at all	1576	21.4%
A little bit	2473	33.7%

Moderately	1598	21.8%
Quite a bit	1306	17.7%
Extremely	393	5.4%

**Table 5:** Association between FI-Lab and various covariates: Columns 3 and 4 contain regression (beta) coefficients and corresponding *p* values from negative binomial regression of FI-Lab (represented as deficit count) on various demographics characteristics.

Variable	Category	Beta	*p* value
Age @ index visit		0.0202	<0.0001

Sex	*Female*	−0.0568	0.0157
	*Male*	*Ref*	—

Race	*Black*	0.1994	<0.0001
	*Hispanic*	0.0528	0.0095
	*Other*	−0.0203	0.7180
	*White*	*Ref*	—

Pre-9/11 occupation	*Construction*	0.0069	0.8290
	*Protective*	0.0158	0.608
	*Other*	0.0360	0.2727
	*CM and IRG* ^*∗*^	*Ref*	—

Education	*Higher than HS*	0.0011	0.7042
	*HS or lower*	*Ref*	—

Enrollment year		−0.0002	0.9536

WTC exposure severity	*Low*	−0.0168	0.5658
	*Medium*	−0.0231	0.2764
	*High/very high*	*Ref*	—

Smoking status @ index visit	*Current*	0.2415	<0.0001
	*Former*	0.0085	0.6557
	*Never*	*Ref*	—

Year of index visit		−0.0065	0.4492

^*∗*^CM: cleaning/maintenance of buildings and grounds; IRG: installation/repair groups (electrical, telecommunications, etc.).

**Table 6:** Prevalence of frailty (stratified by demographic characteristics) within the study sample.

Variable	Strata	% with WTC FI-Lab ≥ 0.25	*p* value (chi-square test)
Age group at index visit	*40–45*	6.7	<0.0001
	*46–50*	8.5	
	*51–55*	11.4	
	*56–60*	16.6	
	*61–65*	21.0	
	*>65*	24.6	

Sex	*Male*	12.6	0.0519
	*Female*	10.6	

Race	*White*	11.3	<0.0001
	*Black*	17.4	
	*Hispanic*	11.6	
	*Other*	10.1	

Pre-9/11 occupation	*Construction*	13.9	<0.0001
	*Protective*	9.8	
	*Other*	15.8	
	*CM and IRG*	13.3	

Education	*Higher than HS*	11.6	0.0096
	*HS or lower*	13.8	

Enrollment year	*2002–2005*	12.5	0.7717
	*2006–2008*	12.1	
	*>2008*	11.9	

WTC exposure severity	*Low*	13.2	0.5677
	*Medium*	12.2	
	*High/very high*	11.8	

Smoking status at index visit	*Current*	20.6	<0.0001
	*Former*	14.1	
	*Never*	10.3	

Year of index visit	*2012*	11.2	0.2378
	*2013*	11.9	
	*2014*	12.7	
	*2015*	14.3	
	*2016*	14.6	
